# The Impact of Ink Composition and Its Physical Properties on the Selected Attributes of 3D-Printed Fruit Purées with Hydrocolloid Molecules

**DOI:** 10.3390/molecules30163394

**Published:** 2025-08-15

**Authors:** Zuzanna Domżalska, Ewa Jakubczyk

**Affiliations:** Department of Food Engineering and Process Management, Institute of Food Sciences, Warsaw University of Life Sciences, 02-776 Warsaw, Poland

**Keywords:** food ink, additive manufacturing, printing, hydrocolloids, rheology, texture, syneresis, extrudability, fidelity, stability

## Abstract

The study aimed to evaluate the influence of ink composition, a blend of blueberry and banana purée with hydrocolloids such as xanthan gum and carrageenan in concentrations ranging from 1 to 4%, on various physical properties. These parameters included dry matter, water activity, density, syneresis index, and rheological and textural attributes of fruit inks. Additionally, the stability of the inks post-printing and after 60 min was examined using image analysis method. Increased hydrocolloid additives from 1 to 4% caused the increase of the viscoelastic modulus G′ and G″, force and extrusion work values extrudability of inks. The stability and fidelity of the inks were enhanced, resulting in a notable reduction in syneresis during storage. The modulus of elasticity exceeded the modulus of viscosity for all ink formulations evaluated, thereby ensuring structural stability. Notably, the formulation comprising 4% xanthan gum and 4% carrageenan exhibited the highest values in both viscoelasticity and extrudability indices, indicating superior performance characteristics within the studied parameters. The shape of the printed objects remained comparable to the designed model over time. Considering the constraints associated with the use of carrageenan, it is possible to attain a comparable effect by utilising reduced concentrations of hydrocolloids. For instance, formulations incorporating 3% xanthan gum in tandem with either 3% carrageenan or 2% carrageenan can achieve similar functionalities. The 3D printing of fruit purées, including blueberries and bananas, represents a significant innovation in personalising food products in terms of consistency. This is particularly relevant for individuals with dysphagia, children, and the elderly.

## 1. Introduction

Additive manufacturing, often called 3D printing, is a transformative process in producing three-dimensional products. 3D printing is a technique that involves creating physical objects from a CAD (Computer-Aided Design) model. There are various processes in which material is deposited layer by layer, allowing the individual layers to join together to form a final 3D object. Additive manufacturing encompasses a range of advanced rapid prototyping techniques, including but not limited to material jetting, binder jetting, extrusion, vat photopolymerization, powder fusion, sheet lamination, and energy deposition [1,2]. 3D printing technology has applications across various industries, including food processing, which is referred to as Food Layered Manufacturing (FLM). The convergence of digital gastronomy and 3D printing technology has facilitated the advancement of FLM techniques [1,3].

Among the different methods for additive manufacturing, the extrusion-based technique is recognised as the most appropriate for 3D food printing applications [4]. This fundamental technique is used in 3D printing, where food material is extruded through a nozzle and layered to create a three-dimensional object [5]. The process consists of four essential steps: preparing materials, extruding ink, depositing it, and forming a 3D structure [6]. In the domain of three-dimensional extrusion printing, the delivery of edible ink is facilitated through a nozzle employing various mechanisms. These mechanisms may include pneumatic pressure, a piston-driven system, or a screw extruder [7]. The predominance of extrusion-based 3D printing can be attributed to its inherent simplicity, cost-effectiveness and versatility in accommodating a diverse range of food materials. The primary raw materials utilised in three-dimensional printing processes include purées, sols, gels, doughs, and emulsions. The formulation of these materials is carefully engineered to optimise their rheological properties, thereby ensuring a consistent and controlled flow through the printer nozzle. This meticulous design is essential for achieving precision in layer deposition and overall structural integrity in the printed object [8].

The materials utilised in the printing process, frequently referred to as “food inks,” comprise a diverse array of ingredients. These ingredients include dairy products, eggs, cheese, chocolate, various oils, plant materials, and meats. This range of components facilitates a broad spectrum of culinary applications and enhances the versatility of food ink formulations [9]. The ink must exhibit sufficient plasticity to flow through the nozzle. Also, it must possess adequate structural stability post-printing to maintain its integrity [7]. The rheological properties of the inks are crucial, as they significantly influence both extrudability and stability of the material during the printing process [10].

Food 3D printing enables the precise tailoring of nutritional profiles to meet the specific dietary needs of individual consumers, but also allows for extensive customisation in terms of dimensions, shape, colour, texture, and flavour [11,12]. The technique can potentially enhance the consumption of fruits and vegetables, particularly among vulnerable populations such as children and seniors, who frequently experience challenges related to swallowing and chewing.

Fruit and vegetables are essential to the human diet, supplying crucial vitamins, carbohydrates, minerals, and bioactive compounds [13]. The regular consumption of fruit and vegetables has been demonstrated to play a pivotal role in the primary prevention of chronic diseases. This phenomenon can be attributed to the bioactive compounds present in these foods and their substantial antioxidant properties [14].

Fruit and vegetables can be used in the 3D printing process. However, they are classified as non-printable raw materials due to their high water content, low protein and fat content, and the possibility of phase segregation [9]. Water, as the primary component of fruit and vegetables, plays a significant role in influencing the viscosity of the ink. This viscosity depends on the specific vegetable raw material used in the formulation. The incorporation of hydrocolloids into non-printable raw materials, including fruits, vegetables, rice, and meat, has been demonstrated to enhance their extrusion capacity [13]. Gelling and binding agents such as xanthan gum, guar gum, locust bean gum, agar or carrageenan can be added to achieve the appropriate structure and stability of inks [9].

Hydrocolloids possess a variety of functional properties, including gelling, thickening, stabilising, and emulsifying. Group of hydrocolloids include, i.a., guar gum, alginate, agar, carrageenan, gellan gum, xanthan gum, gelatine, and modified starches [15]. In food production, using hydrocolloids has become a common practice, primarily aimed at enhancing the final product’s rheological, sensory, and textural characteristics [16]. The composite gels based on peach, gum polysaccharide, and gelatine showed moderate viscosity, good print quality, and structural stability [17]. A comparative study investigated the impact of xanthan gum, konjac gum, and locust bean gum on the rheological properties, textural attributes, moisture content, printability, and sensory characteristics of pea protein-based inks. The experimental concentrations of these hydrocolloids ranged from 0.3 to 0.7% (*w*/*w*). The addition of xanthan gum resulted in a reduction of the melt limit and the viscosity. Conversely, incorporating konjac gum and locust bean gum increased viscosity [18]. Polysaccharide hydrocolloids can form gel structures and be subjected to extrusion moulding under optimal conditions, rendering them viable materials for three-dimensional printing applications. Incorporating an optimal amount of these hydrocolloids can significantly improve the mechanical strength of printed structures and increase the models’ shape stability. This advancement facilitates the production of more complex and precise food gels using 3D printing technology [14,17].

In this study, two selected hydrocolloids were utilised: xanthan gum and carrageenan. These were selected on the basis of their functional properties, plant and microbiological origin, wide range of applications in food technology, and proven effectiveness in food 3D printing applications in numerous studies [19,20,21].

κ-Carrageenan, extracted from red algae, is a sulphated polysaccharide with thermo-reversible properties that forms brittle gels [19,22]. κ-Carrageenan exhibits high water-binding capacity, which is crucial for its applications in food [23]. The viscosity and elasticity of κ-carrageenan are influenced by concentration, pH, and the presence of salts. At higher concentrations, κ-carrageenan transitions from a disordered coil to an ordered helical conformation, forming gels [24]. This hydrocolloid helps in maintaining intestinal homeostasis by positively influencing gut microbiota composition [25]. The advantages of this additive are numerous, encompassing its cost-effectiveness, widespread availability, and biodegradability [26]. However, the acceptable daily intake (ADI) for κ-Carrageenan is 75 mg/kg body weight per day [27].

Xanthan gum, produced by *Xanthomonas campestris*, exhibits significant pseudoplastic behaviour, characterised by its ability to decrease in viscosity under applied shear forces and subsequently recover its viscosity rapidly upon the cessation of such forces [19,22]. The utilisation of xanthan gum in diets for individuals afflicted with dysphagia is of particular significance, given its extensive application in this context due to its efficacious rheological properties, stability against salivary enzymes, and considerable resistance to both acidic and alkaline conditions [28]. This biopolymer has a wide and varied range of applications due to its remarkable physicochemical properties, biodegradability, and non-toxicity [29].

In this study, printing inks were developed based on banana and blueberry purées, with the addition of hydrocolloid mixtures, specifically xanthan gum and carrageenan, in concentrations ranging from 1 to 4%. The ink prepared in this manner was subsequently printed by means of extrusion-based 3D printing. The objective of the present study was to ascertain the impact of ink composition and physical properties on the texture and shape retention of objects printed using 3D technology. The impact of the materials was assessed in terms of physical properties such as dry weight, water activity, density, syneresis, rheological properties (i.e., elasticity and viscosity modulus), and textural properties (i.e., strength and deformation work), as well as dimensional stability of the structures after printing. The printed models were analysed immediately after production and after 60 min of storage in order to assess their durability and compliance with the intended geometric model. The 3D printing of fruit purées, including banana and blueberry, represents a pioneering technique for the fabrication of foodstuffs with customised consistency and form. This technology is particularly useful in the provision of sustenance for individuals suffering from dysphagia, children, and the elderly, for whom conventional products may be difficult to accept due to their texture or appearance. Through the implementation of suitable rheological modifications, it is possible to preserve the nutritional value of fruit while simultaneously enhancing the sensory and functional appeal of food products.

## 2. Results and Discussion

### 2.1. Characteristic of Selected Physical Parameters of Fruit Purée-Based Inks

Selected physical properties of the inks are summarised in Table 1 for the blueberry purée-based inks and Table 2 for the banana purée-based inks. The samples were assigned the following designations: A for blueberry purée-based inks, B for banana purée-based inks. The symbols XG (xanthate gum) and C (carrageenan) are preceded by a number, which refers to the content of the hydrocolloid used.

Syneresis is an undesirable process that separates the liquid phase from semi-solid food systems, including fruit purées, fillings, jams, and jellies. Increased degree of syneresis can lead to a deterioration of the textural characteristics of the product, a decrease in stability during storage and adversely affect consumer sensory perception [30,31]. In this study, the quantitative measurement of syneresis was based on determining the wetted area of the filter paper. In the analysis of blueberry inks, the control sample A_0XG_0C (pure purée) demonstrated the highest level of syneresis, quantified at 448.99% (Table 1). Conversely, the hydrocolloid-containing variants exhibited a substantial reduction in fluid leakage with values ranging from 269.31 to 94.75%. A comparable trend was observed in banana inks, where the control sample B_0XG_0C characterised the highest level of syneresis, 118.03% (Table 2). Furthermore, an increase in the proportions of xanthan gum and carrageenan blends within the formulation correlated with a progressive reduction in leakage area, ultimately achieving minimal values in formulations containing 4% xanthan gum (XG) and 4% carrageenan (C). Ink variants containing banana purée demonstrated a substantial decrease in syneresis index from 67.02 to 30.48% with an increase in gum additions. These values were significantly lower than those observed for variants based on blueberry purée. This difference can be attributed to the different chemical composition of the fruit raw materials used. In contrast to blueberries, bananas contain substantial quantities of starch, which possesses the capacity to absorb water and form gels, particularly in the presence of thickening agents such as hydrocolloids. The starch present in bananas has been shown to enhance the water-binding effect of xanthan gum and carrageenan, thereby contributing to improved water retention [32]. Pant et al. [33] conducted a study on the syneresis of vegetable inks, using a comparable methodology. The study’s findings indicated that variations of pea-based inks containing xanthan gum and carrageenan displayed a significant reduction in syneresis compared to the control ink (which did not include hydrocolloids), which exhibited a syneresis of 80%. However, the inks made from carrot puree and containing only xanthan gum showed a significantly reduced water-wetted area compared to the other variations. Xanthan gum functions as a thickening agent and a mild gelling agent, effectively inhibiting syneresis in this context. Its unique structural properties contribute to the stabilisation of formulations by enhancing viscosity and forming a gel-like consistency, thereby preventing the separation of liquid phases from the solid matrix. Panyayong et al. [34] demonstrated that the addition of hydrocolloids significantly improved the consistency and cohesiveness of the product, concurrently resulting in a noteworthy reduction in syneresis. In a study conducted by Yang et al. [35], the effect of hydrocolloids on kimchi’s seasoning was investigated, revealing a significant reduction in liquid leakage in a concentration-dependent manner. The xanthan gum demonstrated the highest syneresis-reducing efficiency for carrot- and squid-based inks for 3D printing with hydrocolloids [36].

A significant decrease in syneresis was observed in banana-based inks compared to blueberry-based inks, especially in inks without added hydrocolloids. This effect may be due to the fruit’s chemical composition, specifically its water and dry matter content. The dry matter content of the blueberry purée-based inks varied from 13.59 to 20.08%, with statistically significant differences observed among the tested variants (*p* < 0.05) (Table 1). In contrast, the inks produced with banana purée had a significantly higher dry matter content, ranging from 21.94 to 25.05%, with statistically significant differences between samples (Table 2). The highest dry matter content was recorded for samples B_3XG_2C (25.05%) and B_4XG_4C (24.94%). This finding indicates that the incorporation of xanthan gum and carrageenan additives significantly contributes to the enhancement of dry matter concentration in the ink formulation. As noted by Ünal et al. [37], a higher dry matter content can increase the product’s viscosity and water holding capacity. The variations in water content within the inks may also be attributed to differing levels of water binding associated with the incorporated hydrocolloids.

The water activity of the inks ranged from 0.958 to 0.970 (Table 1 and Table 2). The high-water activity observed in the inks can be primarily attributed to the characteristics of the base ingredient of the inks, which consisted of fruit purée, coupled with a minor incorporation of dry matter in the form of a hydrocolloid blend. Increasing the addition of hydrocolloids did not have a statistically significant effect on water activity for inks relative to purée.

Density is a crucial technological parameter that influences both the material distribution within a structure and the final mass of the printed object. Sato et al. [38] suggested that density had a direct impact on mechanical stability and the precision of geometric reproduction. The results showed that the density of blueberry purée-based inks ranged from 1.10 to 1.16 g/cm^3^ (Table 1), while banana inks had density values varying from 1.08 to 1.18 g/cm^3^ (Table 2). The observed variations in density were minimally influenced by the composition of the ink. This can be attributed to the relatively low concentration of hydrocolloids utilised in the formulations, which did not significantly impact the density. Consequently, the density was consistent across all specimens, remaining within a comparable range.

### 2.2. Analysis of the Particle Size Distribution of Fruit Purées

**Figure 1:**
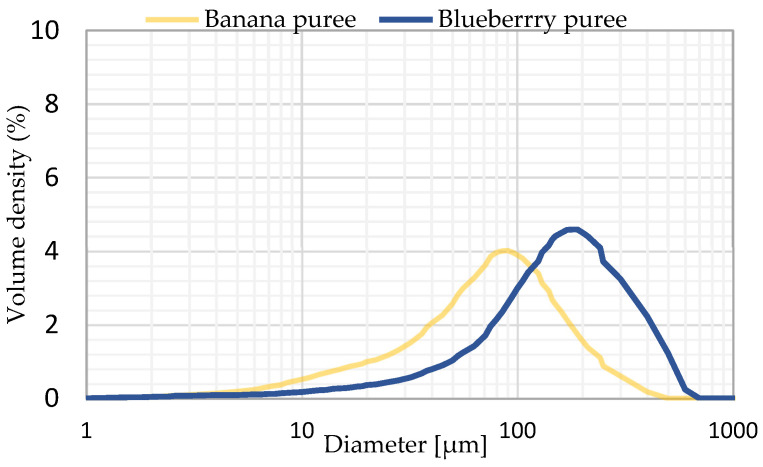
Particle size distribution of blueberry and banana purée.

Particle size distribution curves for blueberry and banana purée are shown in Figure 1. The mean diameter D50 of particles in the blueberry purée was 149.09 µm, but the banana purée exhibited a smaller average diameter of 71.26 µm (Table 3). These differences can be attributed to the intrinsic structural characteristics of the fruit tissues. It was observed that the smaller particles primarily consisted of individual parenchymal cells, while the larger particles were identified as cellular aggregates [38,39].

The dispersion coefficient, commonly referred to as the span, representing the width of the particle size distribution [26], was measured at 1.94 for the blueberry purée and 2.13 for the banana purée. These results indicated that banana purée was characterised by a broader size distribution, suggesting greater particle dimension heterogeneity. According to Eraso-Grisales et al. [40], the particle size distribution values at the 10% level usually correspond to colloidal elements. In contrast, D50 values exceeding 100 µm may indicate the presence of small, coalescing or insoluble fragments. Xing et al. [41] demonstrated D50 values of 265 µm for strawberry pulp, which were reduced to 152.07 µm following ultrasonic treatment. This study showed that particle size had a substantial influence on the sensory characteristics, stability, and rheological properties of pulp. The smaller the particle size of the slurry, the higher its stability.

### 2.3. Rheological Properties of Fruit Purées

The rheological properties of the inks play a critical role in the 3D printing of food materials by extrusion, as these properties determine the material’s ability to reproduce the intended pattern and maintain its shape once deposited [42,43].

**Figure 2:**
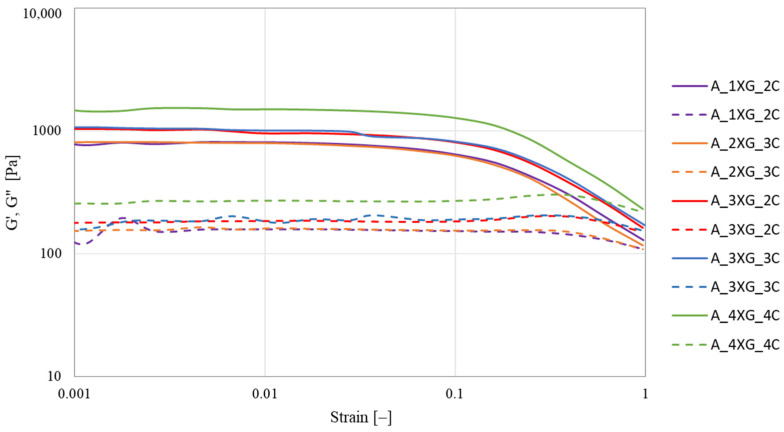
Strain sweep curves of blueberry purée-based ink. Solid lines—G′ and dashed lines—G″.

**Figure 3:**
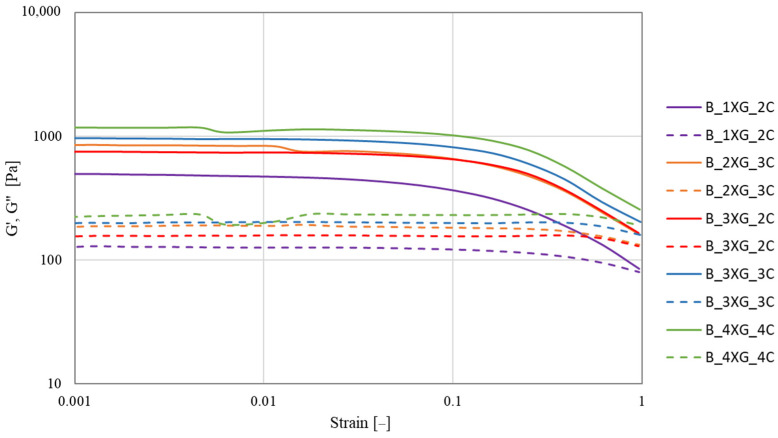
Strain sweep curves of banana purée-based ink. Solid lines—G′ and dashed lines—G″.

The strain sweep was measured within the range from 0.0001 to 1 at a frequency of 1 Hz for the blueberry (Figure 2) and banana (Figure 3) purée-based inks. The analysis of both samples showed a predominance of G′ over G″ (G′ > G″). Liu et al. [44] analysed the rheological properties of surimi with the addition of various hydrocolloids. The results indicated that the G′ of all samples exceeded G″, indicating the presence of a solid-like structure. The incorporation of polysaccharides, including κ-carrageenan (KC), konjac gum (KG), sodium alginate (SA), and guar gum (GG), resulted in an increase of G′. In contrast, the incorporation of xanthan gum (XG) demonstrated an antagonistic effect, resulting in a reduction in the modulus of elasticity and, as a consequence, a compromise in the structural integrity of the gel.

Strain sweep tests were conducted to identify the linear viscoelastic region (LVR), and the moduli were monitored for deviations from linearity. This region extended approximately from γ = 0.001 to 0.08 in most samples. Based on these observations, a fixed strain value of γ = 0.01 was selected for further oscillatory measurements. The sample with blueberry purée A_4XG_4C exhibited the highest resistance to deformation, as indicated by values of G′ (1506.24 Pa) and G″ (270.41 Pa) at a strain of 0.01 (Table 4). Conversely, sample B_4XG_4C demonstrated the highest value of G′ (1123.40 Pa) for the banana-based ink at γ = 0.01 (Table 5). However, G″ was observed to be highest for sample B_3XG_3C (G″ = 204.04 Pa). Statistical analysis revealed a significant increase in G′ and G″ for all blueberry puree-based inks with increasing concentrations of xanthan gum and carrageenan. The xanthan gum solution exhibited pseudoplastic characteristics, enhancing stability and water binding capacity [45].

Kong et al. [46] demonstrated that within the low stress range of 0.001 to 0.1, all composite gels designed for 3D printing comprising proteins, polysaccharides, and incorporating orange juice pouches exhibited a storage modulus (G′) that exceeded the loss modulus (G″). This observation signified their predominantly elastic behaviour, highlighting the structural integrity and potential applications of these materials in various bioprinting processes.

**Figure 4:**
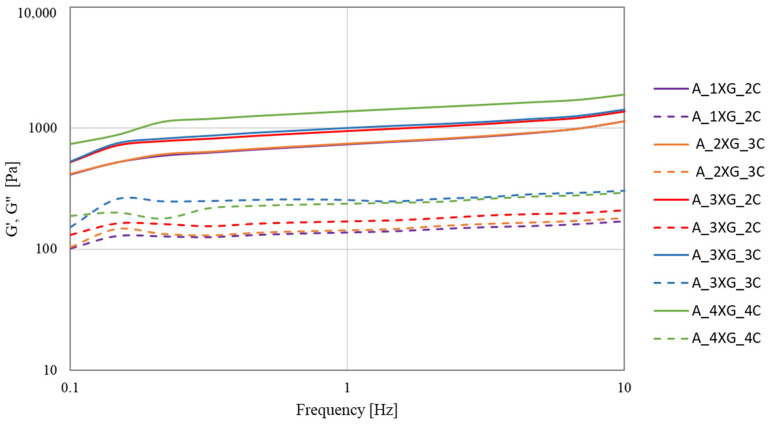
Frequency sweep curves of blueberry purée-based ink. Solid lines—G′ and dashed lines—G″.

**Figure 5:**
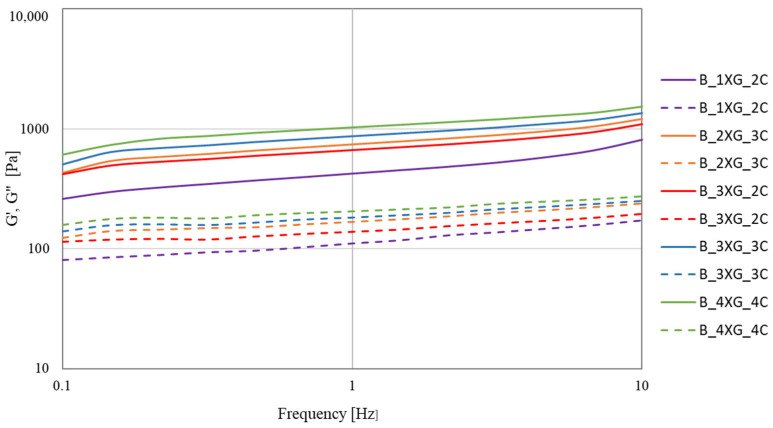
Frequency sweep curves of banana purée-based ink. Solid lines—G′ and dashed lines—G″.

One of the fundamental parameters that characterise the rheological properties of materials is the storage modulus (G′), which determines the resistance of a substance to elastic deformation. It has been demonstrated that higher values of G′ are directly related to the material’s enhanced ability to self-sustain after deposition and greater stiffness and mechanical strength. The second significant parameter is the loss modulus (G″). The frequency sweep test determines the relationship between the test frequency and the material’s moduli (G′) and (G″). The material’s viscoelastic properties can be determined by comparing these values over a range of frequencies [47]. The frequency sweep test was conducted at a constant strain of 0.01 within the 0.1–10 Hz frequency range. This test was carried out to evaluate the rheological properties of the inks. Subsequently, the changes in the storage modules (G′) and loss modulus (G″) as a function of frequency were analysed for blueberry (Figure 4) and banana (Figure 5) purée-based inks.

It was demonstrated that both banana and blueberry purée-based inks showed an increase in G′ and G″ with an increase in frequency. In all samples, G′ > G″ may indicate a dominant elastic character of inks, resulting in good structural stability. Gelatine gels containing different concentrations of apricot pulp (30%, 50% and 70%) showed an increase in both elastic modulus (G′) and loss modulus (G″) with increasing fruit pulp content. Notably, the formulation containing 70% apricot pulp demonstrated the highest resistance to deformation. This observed phenomenon can be attributed to the increased potential for hydrogen bond formation, along with a higher sugar content, which collectively contribute to a more compact and stable gel network, thereby enhancing its resistance to deformation [48].

The highest values of G′ and G″ at 10 Hz (Table 4) were obtained for blueberry puree-based ink A_4XG_4C (G′ = 1891.35 Pa; G″ = 294.45 Pa). The highest stability of banana ink (Table 5) was observed for sample B_4XG_4C (G′ = 1543.56 Pa; G″ = 273.06 Pa). It was established that with increasing concentrations of xanthan gum and carrageenan, the values of G′ and G″ as a function of frequency exhibited a statistically significant increase. In the study conducted by Jiang et al. [49], the impact of incorporating xanthan gum (XG), sodium alginate (SA), and sodium carboxymethylcellulose (CMC-Na) on the characteristics of corn-based composite dough and noodles was examined. The G′ value was higher than G″ for all composite doughs in the frequency range tested (0.1–10 Hz), showing that the composite dough exhibited more elastic behaviour. It was demonstrated that all of these hydrocolloids significantly increased both G′ and G″, while improving the elasticity and viscosity of the dough. The disparities observed in the storage modulus (G′) and loss modulus (G″) values between blueberry and banana inks can be attributed to inherent differences in their chemical compositions, as well as variations in particle size distribution.

Nijdam et al. [50] discovered that G′ values measure the sample’s stiffness or the gel’s strength at rest. This phenomenon significantly impacts the ability to retain structural integrity following the process of 3D printing. In contrast, the parameter G″ represents the energy required to deform the printing ink during its flow. As demonstrated by Zeng et al. [51], rheological tests showed that the predominance of G′ over G″ indicated solid-like behaviour, which minimised the risk of structure collapse after printing. A comprehensive examination of the rheological properties of food inks is paramount for the advancement of 3D printing technologies, particularly in the characterisation of their elastic properties and stability after printing.

It has been observed that the rheological parameters increase with the addition of hydrocolloids, as evidenced by syneresis. It has been established that lower levels of syneresis are associated with higher G′ and G″ values. This is due to a stronger gel network being able to effectively retain water in the structure. The relationship between syneresis and rheological properties has been confirmed in other studies [52].

### 2.4. Extrudability of Fruit Purée-Based Inks

Extrudability is a critical parameter that defines the ease with which a material can be extruded through a nozzle, serving as a fundamental criterion in extrusion-based 3D printing. This property can be quantitatively assessed by measuring the force required for extrusion, providing valuable insights into the material’s performance in additive manufacturing processes [53].

Table 6 showed that the force required for the blueberry ink’s extrusion increased with increasing hydrocolloid addition. The force values ranged from 5.41 N for A_1XG_2C ink to 14.89 N for A_4XG_4C objects. The extrusion work increased from 99.22 to 285.09 mJ for these samples. A similar trend was observed for the banana samples; the extrusion force varied from 7.02 N (B_1XG_2C) to 15.61 N (B_4XG_4C), and the extrusion work ranged from 131.73 to 286.22 mJ (Table 7). In inks with banana and blueberry puree, the samples with the highest concentrations of XG (4%) and C (4%) hydrocolloids demonstrated the highest resistance to extrusion. This could be advantageous for the precision of 3D printing. As demonstrated by the forward extrusion test, variations in the concentrations of xanthan gum and carrageenan exhibited a statistically significant effect on the rheological properties of inks formulated with blueberry and banana purée. Increased force and extrusion work were observed in response to increased hydrocolloid concentrations. This increase in hydrocolloid levels may provide adequate mechanical strength of inks, facilitating the retention of their geometric integrity post-3D printing.

The increased concentration of hydrocolloids present in ink formulations may necessitate additional effort to mitigate the friction occurring between the particles of the purée and between these particles and the container. If this required effort becomes excessive, it can complicate the printing process, potentially resulting in clogged cartridges. Conversely, if the incorporation of hydrocolloids is inadequate, the purée ink may demonstrate ease of extrusion; however, it may exhibit instability post-printing.

It has been established that rheological properties, including but not limited to elastic modulus and viscosity, play a pivotal role in the extrusion process. A rise in G′ was found to be significantly associated with enhanced extrudability. In a study of a multi-component carrageenan–xanthan–starch gel system in extrusion-based additive manufacturing, Liu et al. demonstrated that an increase in elasticity (G′) and the corresponding viscosity (G″) was crucial for efficient extrusion and print accuracy [19].

### 2.5. The Printing Precision and Stability of Printed Objects

Filament fidelity is defined as the capacity of the extruded material to maintain its structural integrity, thereby preventing warping and collapse. Insufficient yield strength can lead to deformation under the material’s weight; consequently, bulking and thickening agents such as food-grade hydrocolloids are incorporated to enhance structural stability [54]. The consistency of layer height during the 3D printing process is a critical factor in ensuring shape stability and the overall success of the printing process [55]. Furthermore, achieving an optimal balance of rheological properties is essential for effective 3D printing. Thus, meticulous calibration of the viscoelastic properties is imperative; it must exhibit sufficient fluidity to facilitate extrusion while maintaining the structural integrity necessary to preserve its shape post-deposition [56].

**Figure 6:**
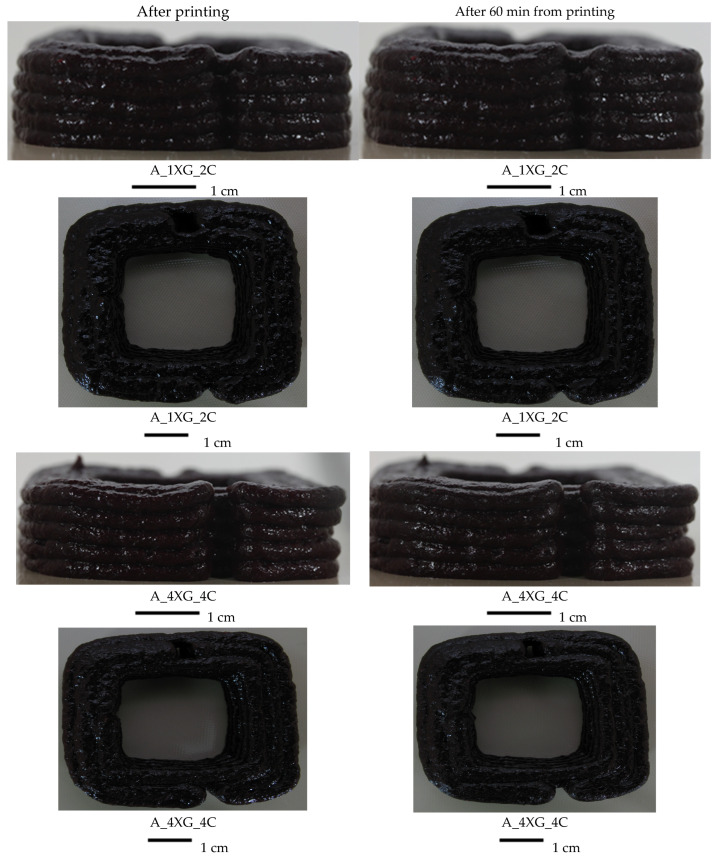
Images of 3D-printed blueberry purée-based cuboids with different concentrations of hydrocolloid after printing and after 60 min of storage.

**Figure 7:**
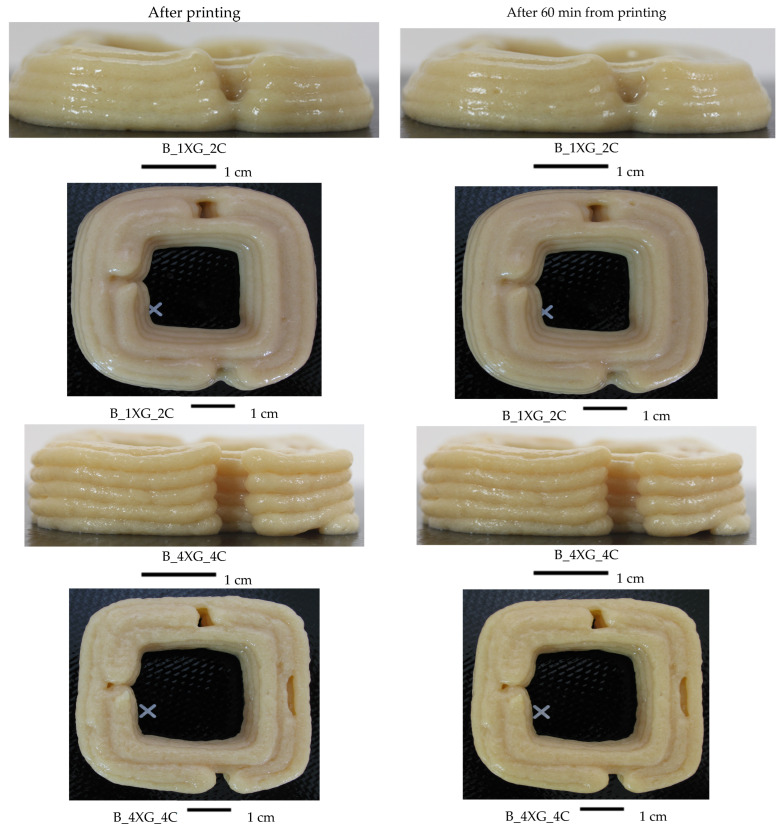
Images of 3D-printed banana purée-based cuboids with different concentrations of hydrocolloid after printing and after 60 min of storage.

As demonstrated in Figure 6 and Figure 7, the printed objects were examined immediately following printing and 60 min thereafter. In the present study, two extreme ink variants were presented for each type of purée based on blueberry (A) and banana (B), with the addition of 1% xanthan gum (XG) and 2% carrageenan (C), and with the addition of 4% XG and 4% C.

The image analysis results for objects printed using inks based on blueberry and banana purées are presented in Table 8 and Table 9, respectively. The cross-sectional area of the printed objects was compared to that of the model cuboid. Additionally, this area was measured 60 min after the printing of the objects, allowing for a comparison with the model’s area. The height of the cuboid was also analysed immediately after printing and again after 60 min. These parameters can provide insights into the reproduction accuracy and object stability following the printing process.

The differences in the printed object’s area after 60 min of storage ranged from 2.3% to 4.2% for the blueberry purée-based inks, indicating minimal change in material structure during the storage (Table 8). Kim et al. [57] demonstrated that products based on vegetable powders (broccoli, spinach, and carrots) exhibited comparable stability in shape and structure when hydrocolloids, including xanthan gum (XG), were incorporated. The study demonstrated a substantial impact of hydrocolloid composition and powder content on the flexibility and stability of printed structures. Products containing 10% powder and XG demonstrated high resolution and geometric stability during printing, effectively suppressing particle swelling and maintaining satisfactory shape. Hydrocolloids with high water-holding capacity (WHC), such as XG, were pivotal in ensuring adequate extrudability and printing stability.

Variant A_1XG_2C displayed the lowest height of the printed object (1.67 cm), the most significant deviation from the reference model, which had a height of approximately 1.8 cm. This result may suggest a transient instability of the material immediately post-printing. After 60 min, both the cross-sectional area and the height of the printed cuboid exhibited a reduction from the initial measurements recorded immediately after printing, indicating that the material may undergo settling over time. The modifications are delineated in Figure 6, which presents lateral and projection images corresponding to two distinct hydrocolloid concentrations (1% XG + 2% C and 4% XG + 4% C). The analysis of the images demonstrated that structures formulated with higher hydrocolloid concentrations exhibited enhanced stability. The study conducted by Dankar et al. [12] also showed that adding hydrocolloids, in this case 1% alginate, into the potato purée had a significant impact on the stability of the final 3D-printed product. The resultant layers exhibited perfect overlap and a smooth surface, and retained their original shape for an extended period following the printing. Chen et al. [58] evaluated the printing properties of inks derived from textural inks of soy protein isolate, incorporating various hydrocolloids. The study showed that inks with xanthan gum exhibited optimal printing properties and the capacity to obtain structural integrity during the printing process.

The differences in printed area after 60 min ranged from −1.6% to 4.9% for the banana purée-based inks (Table 9), indicating minimal change in material structure after storage. It was observed that only sample B_1XG_2C exhibited an increase in object area after 60 min, which may be attributable to material spillage, as depicted in Figure 7, and inadequate water binding by the hydrocolloids. Han et al. [59] observed that the gels had an increased tendency for damage due to shear forces at higher starch concentrations. This process leads to the dissolution of hydrogen bonds, resulting in a reduction of viscosity and concomitant destabilisation of the structure. The stability of the 3D-printed lemon mousses was analysed after printing and after 30 min. Samples with a higher gelatine content demonstrated significantly greater resistance to structural collapse immediately post-printing when contrasted with those with a lower gelatine content. This observation suggests a pronounced tendency towards deformation in the latter group. Incorporating hydrocolloids, such as gelatine, enhanced the firmness and shape stability of the mousses [60].

Similar to the results of blueberry inks, the banana purée-based printed samples with the smallest addition of hydrocolloids had a height of 1.45 cm, reflecting the most significant deviance from the model height (Table 9). As shown by Liu et al. [61], the minimal addition of carrageenan (0.5%) can significantly improve printing accuracy at low printing speeds (12 mm/s), highlighting the importance of precise additive selection.

After 60 min of storage, it was observed that, except for variant B_1XG_2C, there was a reduction in the printed object’s area and height compared to the initial value. This trend indicated the tendency of the material to collapse over time. Fan et al. [62] also reported changes in surface properties and ink particle size as a function of starch content. The variability of the components was necessary for the spatial and textural stability of 3D structures.

The extrusion test yielded significant findings that directly correlated with observations of the 3D printing process. Inks with higher extrusion force and work exhibited superior geometric stability after printing, thereby confirming that the material’s increased viscosity and mechanical strength promote shape retention consistent with the model. Despite their ease of application, samples exhibiting lower extrusion resistance frequently resulted in structural deformation and loss of dimensional accuracy following printing. At the same time, data from rheological tests, including higher values of the elastic modulus (G′) and the predominance of the elastic component over the viscous component (G′ > G″), indicated the gel’s ability to maintain its structure after extrusion.

### 2.6. Texture of Printed Objects

Texture is a physical property of food’s structural and sensory attributes. Texture analysis is crucial in understanding the structural integrity of three-dimensional (3D) printed food products and assessing their corresponding sensory properties [63].

The results of the maximum compressive force (N) and work of deformation (mJ) are shown in Table 10 and Table 11, respectively.

The highest compression force and deformation work for blueberry-based inks were obtained for ink variant A_4XG_4C (5.57 N and 10.15 mJ), but the lowest value of these parameters was recorded for A_1XG_2C ink (3.20 N and 19.93 mJ) (Table 10). The increase in mechanical attributes indicated increased compressive strength of the printed object with higher concentrations of hydrocolloids. The results may indicate a significant relationship between material stability and hydrocolloid addition. In the analysis of banana-based inks, comparable trends were observed (Table 11). An increase in compression force from 3.64 N (B_1XG_2C) to 6.53 N (B_4XG_4C) was recorded with increasing hydrocolloid concentrations. Simultaneously, the compression work increased from 9.66 to 22.72 mJ. Statistical analysis confirmed significant differences (*p* < 0.05) between banana and blueberry inks, substantiating the impact of varying XG and C additives. Notably, the maximal values of the textural parameters were achieved in samples printed with 4% XG and 4% C. Pant et al. [33] observed that, in pea-based inks, the highest hardness was exhibited by an ink containing xanthan gum (0.3% *w*/*w*) and kappa carrageenan (0.3% *w*/*w*).

No statistically significant differences were identified between samples A_3XG_2C and A_3XG_3C, indicating that variations in carrageenan concentration did not markedly influence the product’s texture. In contrast, a comparison of samples A_2XG_3C and A_3XG_2C demonstrated that including xanthan gum exerted a more substantial effect on the hardness parameter than incorporating carrageenan. The higher compressive force and work of deformation values observed in samples containing 4% xanthan gum and 4% carrageenan in both fruit purées were directly related to increased storage modulus (G′) and loss modulus (G″). This indicates stronger cross-linking of the structure. At the same time, these samples exhibited lower syneresis, suggesting more effective water binding within the matrix, thereby increasing resistance to compression further. Banana purées, characterised by a smaller particle size distribution than blueberry purées, exhibited higher compressive strength values at comparable hydrocolloid concentrations.

### 2.7. Principal Components Analysis (PCA)

**Figure 8:**
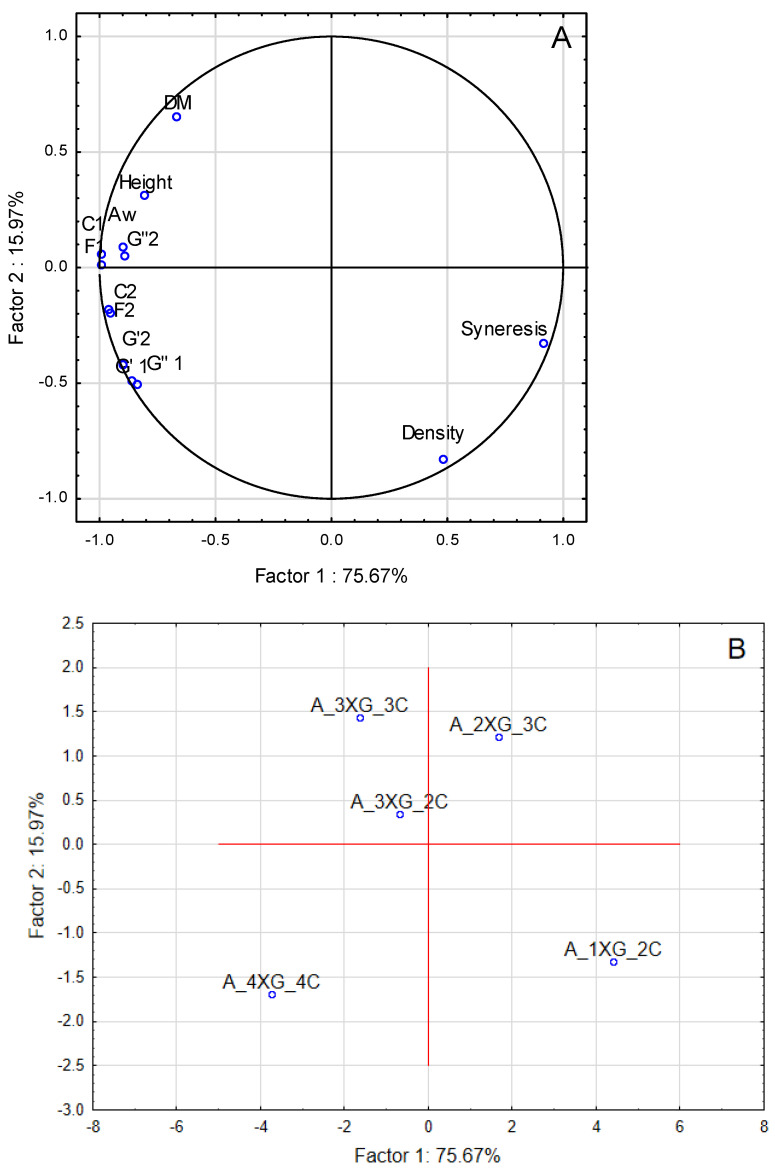
Principal component analysis (PCA) for blueberry purée inks: (**A**) quality variables: Aw—water activity, DM—dry matter, Height—height of 3D-printed object, C1—compression work, F1—compression force, C2—extrusion work, F2—extrusion force, G′1—G′ for ɣ = 0.01. G″1—G″ for ɣ = 0.01, G′2—G′ for f = 10 Hz, G″2—G″ for f = 10 Hz, (**B**) distribution of five variants: A_1XG_2C, A_2XG_3C, A_3XG_2C, A_3XG_3C, A_4XG_4C.

A principal component analysis (PCA) was conducted to ascertain the relationship between the characteristics of the studied inks on blueberry purée base and the addition of different concentrations of a hydrocolloid mixture (xanthan gum and carrageenan). As illustrated in Figure 8A, the initial two components, Factor 1 and Factor 2 explained 91.64% of the total variance of the data. The variables, such as height and dry matter (DM), were strongly correlated with density and syneresis. This observation suggests that these parameters were crucial in the differentiation of samples. The correlation among rheological characteristics (G′1, G″1, G′2, G″2), textural parameters (F1, C1, F2, C2), the precision and stability factor of the printed structures (Height), and water activity (Aw) was evident, indicating a significant influence of hydrocolloid concentration (A_4XG_4C) on these variables.

As illustrated in Figure 8B, the ink samples can be categorised into distinct groups based on the hydrocolloid concentration. Samples with higher concentrations (A_4XG_4C) were distinctly separated from those with lower concentrations (A_1XG_2C), indicating that the hydrocolloid concentration significantly influenced the physical characteristics of the inks and printed objects. Adding hydrocolloids significantly improved the rheological properties of the inks, which translated into greater structural stability and resistance to deformation. Higher values of force and compression work in texture tests also confirmed the enhancement of stability. The result was an improvement in the geometric integrity and durability of the printed objects, which is important for the quality of the final product.

**Figure 9:**
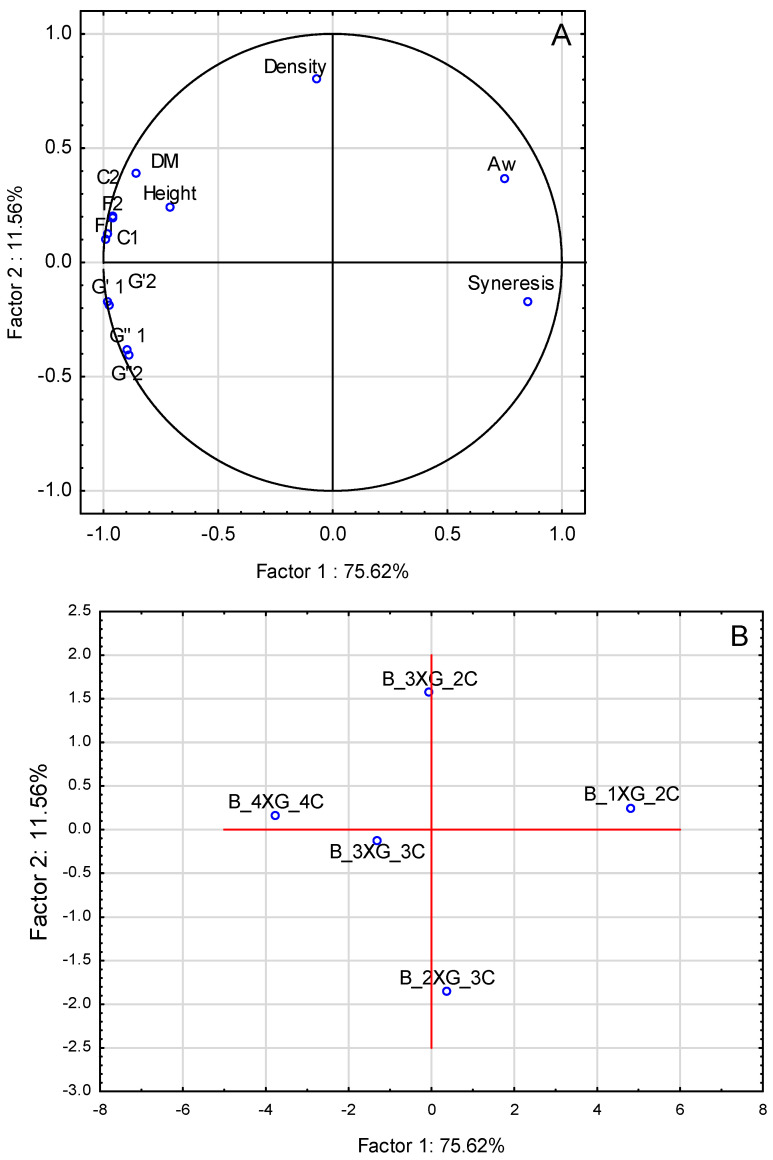
Principal component analysis (PCA) for banana purée inks: (**A**) quality variables: Aw—water activity, DM—dry matter, Height—height of 3D-printed object, C1—compression work, C2—compression force, F1—extrusion work, F2—extrusion force, G′1—G′ for ɣ = 0.01. G″1—G″ for ɣ = 0.01, G′2—G′ for f = 10 Hz, G″2—G″ for f = 10 Hz, (**B**) distribution of five variants: B_1XG_2C, B_2XG_3C, B_3XG_2C, B_3XG_3C, B_4XG_4C.

PCA was conducted to analyse the attributes of banana purée-based inks. The results, illustrated in Figure 9A, indicate that the first two components explained 8.18% of the total variance. In the case of banana puree-based inks (Figure 9A), the variables with the most significant impact on sample differentiation were rheological properties (G′1, G″1, G′2, G″2), textural attributes (F1, C1, F2, C2), a precision of printing, stability factor (height), and dry matter (DM). These parameters were strongly correlated with syneresis. In the PCA analysis, samples with lower syneresis levels were strongly associated with higher dry matter content, higher rheological properties (G′ and G″), and increased textural strength, extrudability, and structural stability (height). This indicates that the reduction in syneresis resulted from strengthening the structural network of hydrocolloids, which promoted water binding in the system. The increased viscosity and elasticity of the mixture, resulting from the higher concentration of hydrocolloids, not only improved the textural characteristics but also facilitated controlled extrusion and increased the geometric stability of the printed structures. Thus, the reduction of liquid phase leakage should be considered a key factor in improving the integrity of the printed object.

In a manner analogous to blueberry inks, a distinct separation of samples (Figure 7) was observed, contingent upon the varying concentrations of the hydrocolloid mixture employed. This observation underscores the notion that alterations in composition can significantly influence the physicochemical properties of the inks. A comparative analysis of the particle size distribution tests alongside the extrusion tests indicated that samples characterised by smaller particle sizes (such as banana purée) exhibited greater challenges during the extrusion process compared to those with larger particle sizes (such as blueberry purée). Furthermore, the relationship between the distribution of particle sizes and the corresponding textural properties was demonstrated in the strength tests conducted on the printed samples, revealing that an increase in the proportion of smaller particles corresponded to an enhancement in strength.

## 3. Materials and Methods

### 3.1. Materials

The study concentrated on the formulation of food inks derived from fruit purées and a range of hydrocolloids. The composition of the mixture included the following ingredients:

- blueberry purée (prepared from the fresh blueberries);
- banana purée (100% banana purée, PURENA, Belfood Sp zo.o., Złoty Potok, Poland);
- κ-carrageenan powder (AGNEX, K. Wierzbicki, Białystok, Poland);
- xanthan gum powder (AGNEX, K. Wierzbicki, Białystok, Poland).

The blueberry purée was obtained using the highbush blueberry (*Vaccinium corymbosum* L.) variety Patriot, which originated from the Jerzy Wilczewski Horticulture Farm in Białousy, Poland. The fruits were placed in a Thermomix^®^ TM6 blender (Vorwerk, Germany) and blended for 30 s at speed 10. Subsequently, the mixture was heated for 6 min at a speed of 3.5 and a temperature of 80 °C. After heating, the blend was processed for 30 s, with the speed gradually increasing from 5 to 9. The purée was passed through a 1.5 mm mesh sieve to standardise the particle size to ensure uniformity.

### 3.2. Methods

#### 3.2.1. Preparation of Fruit-Hydrocolloid Inks

Based on preliminary studies, the composition of the inks was determined and presented in Table 12 and Table 13.

To prepare inks, 500 g of blueberry or banana purée was placed in a 1000 mL beaker, followed by the addition of pre-measured hydrocolloid powders (xanthan gum and carrageenan), calculated based on the concentrations provided in Table 12 and Table 13. The mixture was then blended using a BOSH 600 W blender (New Bern, NC, USA) until a smooth consistency was obtained. The beaker containing the mixture was covered with aluminium foil and placed in a laboratory incubator (Pol-Eko, Wodzisław Śląski, Poland) at 75 °C for 30 min. After this period, the samples were removed from the incubator and permitted to equilibrate to the ambient conditions.

#### 3.2.2. 3D Printing

**Figure 10:**
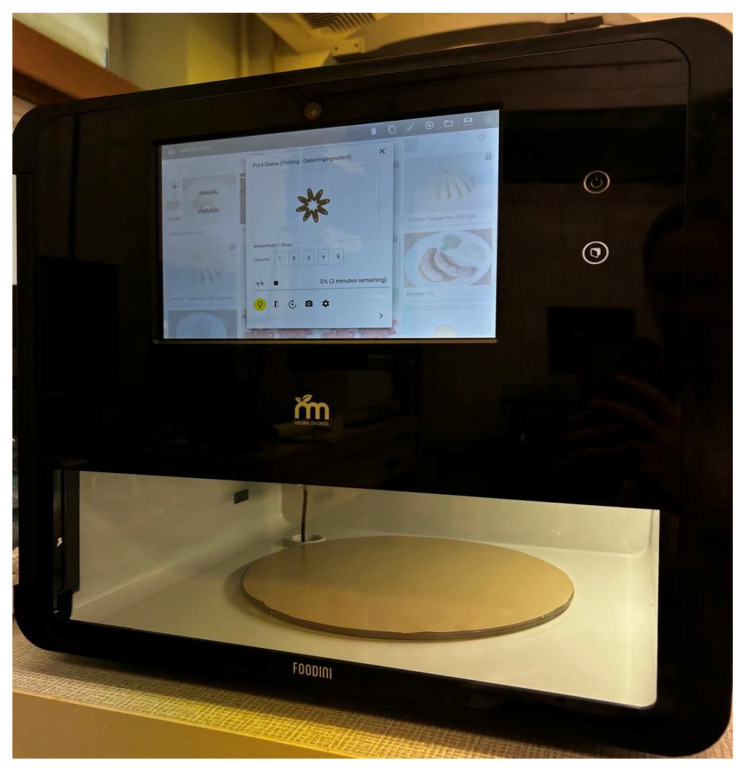
Foodini 3D printer.

**Figure 11:**
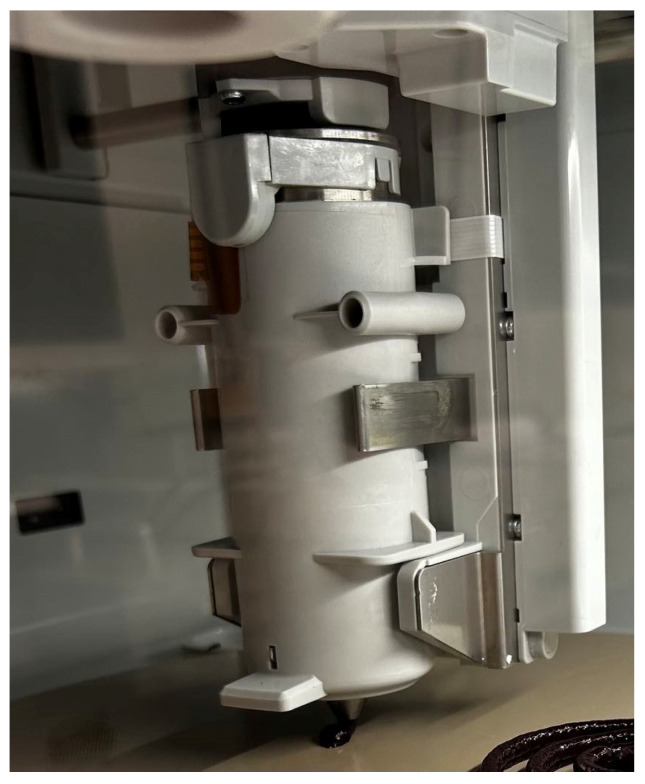
Extrusion printing.

The prepared mixture was transferred into a capsule and fed into an extrusion-based Foodini 3D printer (Natural Machines, Barcelona, Spain). The 3D printer utilised is illustrated in Figure 10, with the technique of extrusion printing illustrated in Figure 11. The hollow cuboid was printed using a circular nozzle of 4 mm diameter. The cuboid consisted of 5 layers. The printing parameters were as follows: a speed of 8000 mm/min, an ingredient flow speed of 1.4, a fill factor of 1%, a first layer hold of 4.2 mm, a first layer nozzle height of 3.5 mm, an ingredient hold of 3 mm, a line thickness of 3.5 mm, and a jump height of 6 mm. The dimensions of the printed model were set as follows: height approximately 1.8 cm, width approximately 5.4 cm.

#### 3.2.3. Water Activity and Dry Matter of Fruit Purées and Inks

The dry matter of fruit purées and inks was measured using the oven-drying method (SUP 65 W/G dryer, Wamed, Warsaw, Poland) according to the procedure applied by Jakubczyk and Jaskulska [64] with some modifications. The samples were mixed with anhydrous sea sand and dried at 105 °C for 24 h. The measurement was repeated two times.

Water activity was measured for banana and blueberry purée and fruit-hydrocolloid inks using an AquaLab Series 3TE device (Decagon Devices, Pullman, WA, USA) with an accuracy of ±0.001. The measurement was performed at 22 °C in triplicate.

#### 3.2.4. Density of Inks

A dish with a volume of 16.7 cm^3^ was used to obtain the density of the inks. The mass of the dish before and after filling it with ink was measured. Jakubczyk et al. described the detailed measurement procedure [65]. The experiment was carried out three times.

#### 3.2.5. Syneresis of Fruit Inks

Syneresis was measured using the filter-paper blotting method described by Pant et al. [33] with some modifications. Syneresis experiments were conducted by placing 15 g of ink at the centre of a filter paper disc with an area of approximately 20 cm^2^. The purées were then flattened to form a circle. The object was left undisturbed for 30 min to facilitate fluid drainage, after which a photograph was taken. The images were then analysed using NIS Elements D software (v. 5.30.00, Nikon, Tokyo, Japan). The leakage area (cm^2^) was determined for each sample. The syneresis index of food inks was calculated from the initial sample area and the sample area after 30 min. Leakage was assessed by calculating the difference between the sample area after 30 min and the initial area, and relating this to the initial value. The result was presented as a percentage increase in area. The test was performed in triplicate.

#### 3.2.6. The Particle Distribution of Fruit Purées

The particle size distribution of banana and blueberry purée was measured based on the principle of laser diffraction using a Cilas 1190 analyser (Cilas, Orléans, France). The puree was suspended in distilled water at an obscuration of 10%. The particle size distribution graph, span, and the median diameter D50 were obtained. The measurement was carried out in triplicate.

#### 3.2.7. Rheological Properties of Fruit Inks

The measurements were conducted using a HAAKE MARS 40 rheometer (Thermo Scientific Inc., Karlsruhe, Germany) with serrated parallel plates with a diameter of 35 mm (P35/Ti/SE) and a gap size of 2 mm. The test course was controlled using Haake RheoWin Manager software v. 4.87.0002 (Thermo Scientific Inc., Karlsruhe, Germany). The rheological properties of fruit-hydrocolloid systems were investigated using the strain sweep test at 1 Hz, with a variable strain γ from 0.0002 to 0.9671 and a temperature of 20 °C to obtain the linear range LVR. The storage modulus G′ (Pa) and the loss modulus G″ (Pa) were determined at strain ɣ = 0.01. Based on the LVR results, a frequency sweep test was carried out with a strain of 0.01 and in the frequency range from 0.1 to 10 Hz at a temperature of 20 °C. The values of G′ and G″ were also determined at a frequency of 10 Hz.

#### 3.2.8. Extrudability of Inks

**Figure 12:**
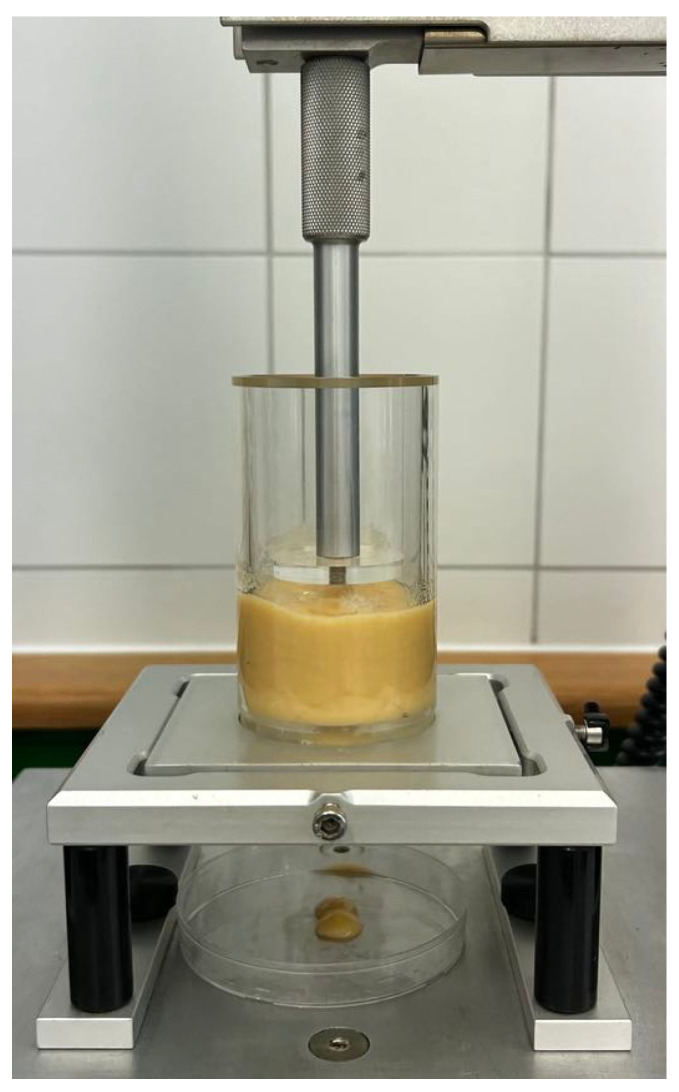
Forward extrusion test of inks.

Forward extrusion tests were conducted using a TA-HD plus texture analyser with a 5 kg load cell (Stable Micro Systems, Surrey, UK). The analyser was equipped with a forward extrusion cell with a base disc of outlet diameter of 5 mm (Figure 12). The container (cell) was carefully filled with ink using a spatula to a constant ink height of 50 mm. The ink was extruded in compression mode using a piston disc with a diameter of 45 mm. The test was carried out with a deformation speed of 1.2 mm/s, pre-travel distance of 12 mm and a target deformation of 20 mm. The force–time curve was obtained, and the maximum extrusion force (N) and extrusion work (mJ) were analysed. The test was carried out 4 times for all inks.

#### 3.2.9. Measurement of Printing Precision and Stability of Printed Objects

**Figure 13:**
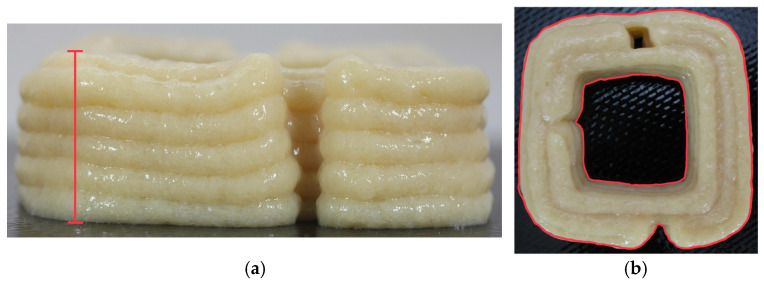
Measured object values: (**a**) height, (**b**) area.

Photographic documentation was conducted using a Canon camera to capture images of the material after the printing process and following 60 min of storage at ambient temperature. The height of the object was recorded in each image presenting the frontal view of the sample (Figure 13a). The projected area after printing and after 60 min was quantified for the images captured from a top-down perspective (Figure 13b).

The measurement process was conducted in triplicate to ensure reliability. Both frontal and overhead images were systematically captured. Subsequently, image analysis was performed using the NIS-elements D3.1 software provided by Nikon Instruments Inc. (Melville, NY, USA). Subsequently, the projected area of the hollow cuboid after printing (A_1_0%) as well as after 60 min of storage (A_1_60%) was measured. These values were referred to the area of the designed 3D model (approximately 30 cm^2^). The differences between A_1_0% and A_1_60% were calculated. The height of the 3D-printed object and after storage (60 min) was also measured.

#### 3.2.10. Evaluation of Textural Properties of Printed Objects

The texture of the printed objects was investigated using a TA-HD plus texture analyser (Stable Micro Systems, Surrey, UK). A compression test was performed at a constant speed of 1 mm/s at a strain of 50% and a threshold value of force ±0.05 N. The measurement data were recorded using Exponent software v. 4.0.13.0 (Stable Micro Systems, Surrey, UK). The test was carried out in seven repetitions. The maximum force of deformation test (N) and compression work (mJ) were analysed.

#### 3.2.11. Statistical Analysis

Statistica v 13.3 software (StatSoft Inc., Tulsa, OK, USA) was used to perform one-way analysis of variance (ANOVA) and Tukey’s test at the 95% significance level. The results were presented as mean ± standard deviation. Additionally, principal component analysis (PCA) was also carried out to identify the most important physical attributes of inks and printed objects.

## 4. Conclusions

The results showed that adding hydrocolloids in the concentration range of 1 to 4%, including xanthan gum and carrageenan, to fruit purée-based inks affected the material’s rheological properties, texture, and stability. Increasing the hydrocolloid content had no significant effect on water activity or dry matter content, due to the high-water content of the fruit purée. The density of the inks was found to be similar across all treatments, irrespective of the type of fruit purée and hydrocolloid content. This finding suggests that the ink’s composition exerted a negligible influence on this particular parameter. The viscoelasticity modules for all the inks that were tested, both blueberry and banana purée-based, were characterised by a clear predominance of the elasticity module over the viscosity modules. The predominance of the elastic modulus may have contributed to the ink’s enhanced extrudability and shape retention after printing, thereby enabling the creation of stable shapes. The examination of fidelity and stability in 3D-printed objects incorporating blueberry and banana purée revealed that increased concentrations of hydrocolloids significantly enhanced the conformity of the printed shapes to their intended models. Ink syneresis studies showed that increasing the concentration of hydrocolloid particles reduced leakage during storage, which was crucial for maintaining the stability of printed objects. Increasing the concentration of hydrocolloids can enhance the mechanical strength of inks; however, an excessive amount may impede the 3D printing process by increasing extrusion resistance. Increasing the concentration of xanthan gum and carrageenan improved the mechanical properties of the inks, with xanthan gum having a more substantial effect on texture than carrageenan. It can be concluded that variants based on blueberry puree, as well as banana purée with 4% xanthan gum and 4% carrageenan addition, are suitable for 3D printing applications, as they show optimal rheological and textural properties, minimal syneresis, and well-defined structural parameters. These characteristics, upon completion of the printing process, were found to be consistent with the predetermined model. However, printing ink compositions were used with the lowest possible level of additives necessary to achieve the desired technological properties. Hydrocolloid mixtures containing 3% xanthan gum and 3% carrageenan, as well as 3% xanthan gum and 2% carrageenan, proved to be optimal for 3D printing and suitable for consumption. This product is particularly suitable for individuals with difficulties in consuming solid foods, such as children, the elderly, or patients with dysphagia and, due to the use of plant-based hydrocolloids, is also appropriate for vegetarian and vegan diets. The effectiveness of hydrocolloids depends on the composition of the fruit, which highlights the need to adjust formulations based on the type of raw material used.

## Figures and Tables

**Table 1:** Selected physical parameters of blueberry purée-based inks.

Ink Variant	A_0XG_0C	A_1XG_2C	A_2XG_3C	A_3XG_2C	A_3XG_3C	A_4XG_4C
Syneresis [%]	448.99 ± 33.14 ^c^	269.31 ± 26.48 ^b^	170.85 ± 20.60 ^a^	105.03 ± 3.41 ^a^	107.80 ± 12.00 ^a^	94.75 ± 11.74 ^a^
Dry matter (%)	13.59 ± 0.67 ^a^	15.68 ± 0.14 ^a^	20.08 ± 1.36 ^b^	19.75 ± 0.29 ^b^	19.88 ± 0.06 ^b^	19.22 ± 0.10 ^b^
Water activity	0.970 ± 0.007 ^a^	0.960 ± 0.007 ^a^	0.960 ± 0.004 ^a^	0.963 ± 0.004 ^a^	0.965 ± 0.003 ^a^	0.964 ± 0.004 ^a^
Density [g/cm^3^]	1.10 ± 0.01 ^a^	1.16 ± 0.02 ^b^	1.13 ± 0.02 ^ab^	1.14 ± 0.01 ^ab^	1.12 ± 0.02 ^ab^	1.14 ± 0.03 ^ab^

Different letters (in rows) indicate statistical differences between groups (*p* < 0.05).

**Table 2:** Selected physical parameters of banana purée-based inks.

Ink Variant	B_0XG_0C	B_1XG_2C	B_2XG_3C	B_3XG_2C	B_3XG_3C	B_4XG_4C
Syneresis [%]	118.03 ± 32.10 ^b^	67.02 ± 14.21 ^ab^	50.99 ± 9.80 ^a^	36.64 ± 10.36 ^a^	32.42 ± 7.78 ^a^	30.48 ± 9.62 ^a^
Dry matter (%)	21.94 ± 0.80 ^a^	23.02 ± 0.64 ^ab^	23.95 ± 0.54 ^ab^	25.05 ± 0.85 ^b^	24.50 ± 0.20 ^ab^	24.94 ± 0.84 ^b^
Water activity	0.960 ± 0.00 ^a^	0.963 ± 0.003 ^a^	0.960 ± 0.003 ^a^	0.962 ± 0.003 ^a^	0.962 ± 0.002 ^a^	0.958 ± 0.005 ^a^
Density [g/cm^3^]	1.18 ± 0.02 ^b^	1.15 ± 0.03 ^b^	1.08 ± 0.02 ^a^	1.15 ± 0.03 ^ab^	1.14 ± 0.03 ^ab^	1.16 ± 0.03 ^b^

Different letters (in rows) indicate statistical differences between groups (*p* < 0.05).

**Table 3:** Particle size distribution parameters of blueberry and banana purée.

Purée Variant	Blueberry	Banana
Diameter D50, μm	149.09 ± 0.68	71.26 ± 0.11
Span	2.13 ± 0.00	1.94 ± 0.01

**Table 4:** Selected rheology parameters of blueberry purée-based ink.

Ink Variant	A_1XG_2C	A_2XG_3C	A_3XG_2C	A_3XG_3C	A_4XG_4C
G′ at ɣ = 0.01, Pa	789.41 ± 24.17 ^a^	796.02 ± 70.07 ^a^	964.91 ± 129.86 ^a^	1010.10 ± 113.86 ^a^	1506.24 ± 58.02 ^b^
G″ at ɣ = 0.01, Pa	151.58 ± 10.10 ^a^	161.80 ± 12.44 ^a^	186.07 ± 12.85 ^a^	178.70 ± 39.73 ^a^	270.41 ± 6.18 ^b^
G′ at 10 Hz, Pa	1118.28 ± 48.21 ^a^	1143.93 ± 78.26 ^a^	1376.06 ± 46.20 ^b^	1395.644 ± 152.29 ^b^	1891.35 ± 38.09 ^c^
G″ at 10 Hz, Pa	162.74 ± 13.30 ^a^	180.80 ± 14.64 ^ab^	209.94 ± 10.18 ^b^	214.7636 ± 33.33 ^b^	294.45 ± 4.37 ^c^

Different letters (in rows) indicate statistical differences between groups (*p* < 0.05).

**Table 5:** Selected rheology parameters of banana purée-based ink.

Ink Variant	B_1XG_2C	B_2XG_3C	B_3XG_2C	B_3XG_3C	B_4XG_4C
G′ at ɣ = 0.01, Pa	472.62 ± 17.98 ^a^	835.98 ± 40.37 ^b^	745.89 ± 105.56 ^b^	954.55 ± 51.52 ^bc^	1123.40 ± 167.55 ^c^
G″ at ɣ = 0.01, Pa	125.63 ± 3.73 ^a^	189.10 ± 14.61 ^a^	158.77 ± 23.00 ^a^	204.04 ± 11.52 ^a^	203.39 ± 71.04 ^a^
G′ at 10 Hz, Pa	815.14 ± 16.55 ^a^	1213.26 ± 68.90 ^b^	1096.70 ± 132.39 ^ab^	1359.28 ± 57.64 ^bc^	1543.56 ± 191.08 ^c^
G″ at 10 Hz, Pa	172.68 ± 3.94 ^a^	239.82 ± 17.42 ^bc^	195.40 ± 27.06 ^ab^	251.70 ± 15.27 ^bc^	273.06 ± 38.46 ^c^

Different letters (in rows) indicate statistical differences between groups (*p* < 0.05).

**Table 6:** Extrudability parameters of blueberry purée-based inks.

Ink Variant	A_1XG_2C	A_2XG_3C	A_3XG_2C	A_3XG_3C	A_4XG_4C
Force, N	5.41 ± 0.22 ^a^	7.69 ± 0.62 ^b^	11.79 ± 0.62 ^d^	10.15 ± 0.27 ^c^	14.89 ± 0.56 ^e^
Extrusion work, mJ	99.22 ± 4.08 ^a^	147.21 ± 11.92 ^b^	225.75 ± 11.87 ^d^	194.45 ± 5.08 ^c^	285.09 ± 10.72 ^e^

Different letters (in rows) indicate statistical differences between groups (*p* < 0.05).

**Table 7:** Extrudability parameters of banana purée-based inks.

Ink Variant	B_1XG_2C	B_2XG_3C	B_3XG_2C	B_3XG_3C	B_4XG_4C
Force, N	7.02 ± 0.27 ^a^	9.610 ± 0.23 ^b^	11.51 ± 0.20 ^c^	11.69 ± 0.41 ^c^	15.61 ± 0.50 ^d^
Extrusion work, mJ	131.73 ± 4.51 ^a^	176.26 ± 4.22 ^b^	211.01 ± 3.61 ^c^	214.28 ± 7.50 ^c^	286.22 ± 9.15 ^d^

Different letters (in rows) indicate statistical differences between groups (*p* < 0.05).

**Table 8:** Fidelity and stability parameters of 3D-printed blueberry purée.

Structural Parameters	A_1XG_2C	A_2XG_3C	A_3XG_2C	A_3XG_3C	A_4XG_4C
Changes of projected area of 3D object in comparison to the model, after printing, A_1_0%	15.1 ± 0.5 ^a^	15.4 ± 0.2 ^a^	12.2 ± 0.2 ^b^	8.0 ± 0.2 ^d^	11.0 ± 0.6 ^c^
Changes of projected area of 3D object in comparison to the model, after 60 min from printing, A_1_60, %	12.8 ± 0.4 ^a^	12.9± 0.4 ^a^	9.4 ± 0.4 ^b^	4.2 ± 0.5 ^d^	6.7 ± 0.5 ^c^
The difference A_1_0-A_1_60, %	2.3 ± 0.8 ^a^	2.4 ± 0.6 ^a^	2.9 ± 0.4 ^a^	3.9 ± 0.6 ^a^	4.2 ± 1.0 ^a^
Height of 3D-printed object, cm	1.67 ± 0.01 ^a^	1.69 ± 0.01 ^a^	1.82 ± 0.02 ^b^	1.88 ± 0.01 ^c^	1.80 ± 0.00 ^b^
Height of 3D-printed object after 60 min, cm	1.64 ± 0.00 ^a^	1.63 ± 0.01 ^a^	1.72 ± 0.06 ^ab^	1.83 ± 0.04 ^b^	1.77 ± 0.05 ^b^

Different letters (in rows) indicate statistical differences between groups (*p* < 0.05).

**Table 9:** Fidelity and stability parameters of 3D-printed banana purée.

Structural Parameters	B_1XG_2C	B_2XG_3C	B_3XG_2C	B_3XG_3C	B_4XG_4C
Changes of projected area of 3D object in comparison to the model, after printing, A_1_0 %	23.3 ± 0.2 ^a^	11.1 ± 0.3 ^c^	10.2 ± 0.2 ^d^	11.0 ± 0.1 ^c^	14.8 ± 0.3 ^b^
Changes of projected area of 3D object in comparison to the model, after 60 min from printing, A_1_60, %	25.0 ± 0.7 ^a^	8.1 ± 0.4 ^c^	6.5 ± 0.2 ^d^	6.1 ± 0.2 ^d^	11.0 ± 0.1 ^b^
The difference A_1_0-A_1_60, %	1.6 ± 0.7 ^d^	2.9 ± 0.6 ^c^	3.7 ± 0.3 ^b^	4.9 ± 0.2 ^a^	3.7 ± 0.3 ^b^
Height of 3D-printed object, cm	1.45 ± 0.01 ^d^	1.70 ± 0.01 ^b^	1.84 ± 0.00 ^a^	1.62 ± 0.03 ^c^	1.76 ± 0.01 ^b^
Height of 3D-printed object after 60 min, cm	1.37 ± 0.04 ^d^	1.62 ± 0.02 ^b^	1.56 ± 0.01 ^c^	1.71 ± 0.01 ^a^	1.70 ± 0.01 ^a^

Different letters (in rows) indicate statistical differences between groups (*p* < 0.05).

**Table 10:** Selected texture parameters of printed objects based on blueberry purée.

Ink Variant	A_1XG_2C	A_2XG_3C	A_3XG_2C	A_3XG_3C	A_4XG_4C
Force, N	3.20 ± 0.14 ^a^	4.21 ± 0.09 ^b^	4.65 ± 0.01 ^c^	4.82 ± 0.31 ^c^	5.57 ± 0.31 ^d^
Compression work, mJ	10.15 ± 0.78 ^a^	14.53 ± 0.66 ^b^	16.65 ± 0.71 ^c^	17.20 ± 0.90 ^c^	19.93 ± 1.98 ^d^

Different letters (in rows) indicate statistical differences between groups (*p* < 0.05).

**Table 11:** Selected texture parameters of printed objects based on banana purée.

Ink Variant	B_1XG_2C	B_2XG_3C	B_3XG_2C	B_3XG_3C	B_4XG_4C
Force, N	3.64 ± 0.36 ^a^	4.98 ± 0.39 ^b^	5.43 ± 0.20 ^bc^	5.74 ± 0.58 ^c^	6.53 ± 0.33 ^d^
Compression work, mJ	9.66 ± 0.47 ^a^	16.34 ± 0.99 ^b^	18.91 ± 0.94 ^bc^	19.44 ± 2.38 ^c^	22.72 ± 2.43 ^d^

Different letters (in rows) indicate statistical differences between groups (*p* < 0.05).

**Table 12:** Composition of prepared inks based on blueberry purée.

Variants of Blueberry (A) Inks	Addition of Xanthan Gum (XG) [%]	Addition of Carrageenan (C) [%]
A_0XG_0C	0.0	0.0
A_1XG_2C	1.0	2.0
A_2XG_3C	2.0	3.0
A_3XG_2C	3.0	2.0
A_3XG_3C	3.0	3.0
A_4XG_4C	4.0	4.0

**Table 13:** Composition of prepared inks with banana purée.

Variants of Banana (B) Inks	Addition of Xanthan Gum (XG) [%]	Addition of Carrageenan (C) [%]
B_0XG_0C	0.0	0.0
B_1XG_2C	1.0	2.0
B_2XG_3C	2.0	3.0
B_3XG_2C	3.0	2.0
B_3XG_3C	3.0	3.0
B_4XG_4C	4.0	4.0

## Data Availability

The original contributions presented in the study are included in the article material. Further inquiries can be directed to the corresponding authors.
